# HER2 and ESR1 mRNA expression levels and response to neoadjuvant trastuzumab plus chemotherapy in patients with primary breast cancer

**DOI:** 10.1186/bcr3384

**Published:** 2013-02-07

**Authors:** Carsten Denkert, Jens Huober, Sibylle Loibl, Judith Prinzler, Ralf Kronenwett, Silvia Darb-Esfahani, Jan C Brase, Christine Solbach, Keyur Mehta, Peter A Fasching, Bruno V Sinn, Knut Engels, Mattea Reinisch, Martin-Leo Hansmann, Hans Tesch, Gunter von Minckwitz, Michael Untch

**Affiliations:** 1 Institute of Pathology, Charité-Universitätsmedizin Berlin, Charitéplatz 1, D-10117 Berlin, Germany; 2 Breast Cancer Center, Heinrich-Heine-University of Düsseldorf, Universitätsstr.1 Düsseldorf, Germany; 3 Breast Center, Kantonsspital St. Gallen, Rorschacher Strasse 95, CH-9007, Switzerland; 4 German Breast Group, Martin-Behaim-Str. 12, D-63263 Neu-Isenburg, Germany; 5 Department of Internal Medicine, Heinrich-Heine-University of Düsseldorf, Universitätsstr.1 Düsseldorf, Germany; 6 Sividon Diagnostics, Nattermannallee 1, D-50829 Cologne, Germany; 7 Siemens Healthcare Diagnostics, Cologne, Nattermannallee 1, D-50829 Germany; 8 Frauenklinik, Johann-Wolfgang-Goethe-Universität, Theodor-Stern-Kai 7, D-60590 Frankfurt am Main, Germany; 9 Department of Gynecology and Obstetrics, University Hospital Erlangen, Friedrich-Alexander University Erlangen Nuremberg, Universitätssstr. 21, D-91054 Erlangen, Germany; 10 Senckenbergisches Institut für Pathologie, Johann-Wolfgang-Goethe-Universität, Theodor-Stern-Kai 7, D-60590 Frankfurt am Main, Germany; 11 Onkologische Gemeinschaftspraxis am Bethanien-Krankenhaus, Im Prüfling 17-19, D-60389 Frankfurt am Main, Germany; 12 Helios Klinikum Berlin-Buch, Schwanebecker Chaussee 50, D-13125 Berlin, Germany

## Abstract

**Introduction:**

Recent data suggest that benefit from trastuzumab and chemotherapy might be related to expression of HER2 and estrogen receptor (ESR1). Therefore, we investigated HER2 and ESR1 mRNA levels in core biopsies of HER2-positive breast carcinomas from patients treated within the neoadjuvant GeparQuattro trial.

**Methods:**

HER2 levels were centrally analyzed by immunohistochemistry (IHC), silver *in situ *hybridization (SISH) and qRT-PCR in 217 pretherapeutic formalin-fixed, paraffin-embedded (FFPE) core biopsies. All tumors had been HER2-positive by local pathology and had been treated with neoadjuvant trastuzumab/ chemotherapy in GeparQuattro.

**Results:**

Only 73% of the tumors (158 of 217) were centrally HER2-positive (cHER2-positive) by IHC/SISH, with cHER2-positive tumors showing a significantly higher pCR rate (46.8% vs. 20.3%, *P *<0.0005). HER2 status by qRT-PCR showed a concordance of 88.5% with the central IHC/SISH status, with a low pCR rate in those tumors that were HER2-negative by mRNA analysis (21.1% vs. 49.6%, *P *<0.0005). The level of HER2 mRNA expression was linked to response rate in ESR1-positive tumors, but not in ESR1-negative tumors. HER2 mRNA expression was significantly associated with pCR in the HER2-positive/ESR1-positive tumors (*P *= 0.004), but not in HER2-positive/ESR1-negative tumors.

**Conclusions:**

Only patients with cHER2-positive tumors - irrespective of the method used - have an increased pCR rate with trastuzumab plus chemotherapy. In patients with cHER2-negative tumors the pCR rate is comparable to the pCR rate in the non-trastuzumab treated HER-negative population. Response to trastuzumab is correlated to HER2 mRNA levels only in ESR1-positive tumors. This study adds further evidence to the different biology of both subsets within the HER2-positive group.

Introduction The human epidermal growth factor receptor 2 (HER2) is the prototype of a predictive biomarker for targeted treatment [1-8]. International initiatives have established the combination of immunohistochemistry (IHC) and *in situ *hybridization as the current gold standard [9,10]. As an additional approach determination of HER2 mRNA expression is technically feasible in formalin-fixed paraffin-embedded (FFPE) tissue [11-13]. Crosstalk between the estrogen receptor (ER) and the HER2 pathway has been suggested based on cell culture and animal models [14]. Consequently, the 2011 St Gallen panel has pointed out that HER2-positive tumors should be divided into two groups based on expression of the ER [15].

A retrospective analysis of the National Surgical Adjuvant Breast and Bowel Project (NSABP) B31 study has suggested that mRNA levels of HER2 and ESR1 might be relevant for the degree of benefit from adjuvant trastuzumab. By subpopulation treatment effect pattern plot (STEPP) analysis in ER-positive tumors, benefit from trastuzumab was shown to be restricted to those with higher levels of HER2 mRNA (S Paik, personal communication, results summarized in [15]).

In our study we evaluated this hypothesis in the neoadjuvant setting in a cohort of 217 patients from the neoadjuvant GeparQuattro trial [5]. All patients had been HER2- positive by local pathology assessment and had received 24 to 36 weeks of neoadjuvant trastuzumab plus an anthracycline/taxane-based chemotherapy. For central evaluation we used three different methods, HER2 IHC, and HER2 silver *in situ *hybridization (SISH), as well as measurement of HER2 mRNA by quantitative real-time (qRT)-PCR [11].

The primary objective of this analysis was to investigate if pathological complete response (pCR) rate in HER2-positive breast cancer would depend on the level of HER2 mRNA expression, with a separate analysis for HR-positive and -negative tumors. Central evaluation of the HER2 status showed that 27% of the tumors with HER2 overexpression by local pathology were HER2-negative. This enabled us to compare response rates in patients with HER2-positive and -negative tumors as a secondary objective.

## Materials and methods

### Study population

The multicenter neoadjuvant phase III GeparQuattro trial (NCT 00288002) recruited a total of 1,509 patients between August 2005 and November 2006. Treatment was four cycles of epirubicin/cyclophosphamide followed by four cycles of docetaxel with or without capecitabine [16]. Patients with HER2-positive tumors (*n *= 445, based on local assessment) received trastuzumab/chemotherapy [5]. FFPE pretherapeutic core biopsies were collected, after written informed consent as part of the prospectively planned collection of biomaterials. Ethical committee approval was obtained for all centers participating in the clinical studies and from the institutional review board of the Charité hospital. A complete pathological response was defined as the pathologically confirmed absence of residual invasive tumor in breast and lymph nodes at surgery. This definition was prospectively defined for this biomarker study before the start of the statistical evaluation in concordance with previous studies [12,17].

Inclusion criteria for the use of biomaterials were the availability of tissue samples from pretherapeutic core biopsies with at least 30% tumor tissue and successful RNA isolation. IHC (rabbit polyclonal anti-HER2 antibody, Clone A0485, DakoCytomation, Hamburg, Germany) and SISH (Inform-SISH system, Ventana, Tucson, AZ, USA) were stained on the Ventana Discovery Autostainer. IHC was performed on 217 cases using large sections, while SISH was performed on a tissue-microarray (TMA) for those 156 cases with available tissue on the TMA. A centrally confirmed HER2-positive status was defined as either IHC 3+ or IHC 2+/SISH ratio >2.0 [9,10]. The study was reported in concordance with the reporting recommendations for tumor marker prognostic studies (REMARK) criteria [18,19].

### Sample preparation and RNA extraction

RNA was isolated from 10-µm FFPE sections using the VERSANT Tissue Preparation System (Siemens Healthcare Diagnostics, Tarrytown, NY, USA) as described earlier [20-22]. Samples had sufficient RNA if the mean of cycle threshold (Ct) values of three reference genes *CALM2, OAZ1 *and *RPL37A *was below 30.6. DNA contamination was assessed by HBB gene-specific qPCR; Ct values above 36 were required.

### Gene expression analysis using quantitative RT-PCR

Primer and probe sequences have been described elsewhere [23]. qRT-PCR was performed in triplicate using the SuperScript III PLATINUM One-Step qRT-PCR System with ROX (Invitrogen, Karlsruhe, Germany) in an Agilent Mx3005 (Agilent, Böblingen, Germany) with 30 minutes at 50°C, 2 minutes at 95°C followed by 40 cycles of 15 sec at 95°C and 30 sec at 60°C. Relative expression levels of genes of interest (GOI) were calculated as ΔCt values as follows:

$$\text{ΔCt}=\text{2}0-\left({\text{Ct}}_{\text{GOI}}-{\text{Ct}}_{\left(\text{mean of RPL37A},\text{ CALM2},\text{ OAZ1}\right)}\right).$$

No-template controls as well as a standardized reference RNA (Stratagene qPCR Human Reference Total RNA, Agilent Technologies, Waldbronn, Germany) were used as controls.

ESR1 and HER2 cutoff values were predefined based on previous studies [11,20,24]. Since different platforms, normalization strategies and primer/probe lots had been used a constant target-specific shift in Ct values between previous and current assay conditions occurred. The cutoffs from the published previous studies were therefore transformed by addition of an offset. The offsets for ESR1 and HER2 were predetermined for this study by reassessment of the 167 samples from the previous study using the new assay conditions, resulting in the cutoffs of 19.5 for HER2 mRNA and 13.8 for ESR1 mRNA. These cutoffs are therefore numerically different, but identical with regard to mRNA levels to the previously published cutoffs. Trained laboratory personnel performed all PCR assays with predefined protocols. All analyses (including the central pathology review) were performed blinded to the clinical data.

### Statistical evaluation

Statistical analysis was performed using SPSS version 19.0 (IBM, Armonk, NY, USA). The probability of pCR as a function of gene expression parameters was determined by univariate logistic regression analysis. The two-sided Fisher´s exact test was used for comparison of pCR rates. STEPP analysis was performed as suggested [25,26] using a sliding window approach of 40 samples with an overlap between windows of 10 cases. pCR rates and mean HER2 mRNA levels were calculated separately for each window for all cases, as well as the two subgroups based on ESR1 mRNA expression.

### Results

### Baseline clinical data, consort statement and central evaluation of HER2

**Figure 1:**
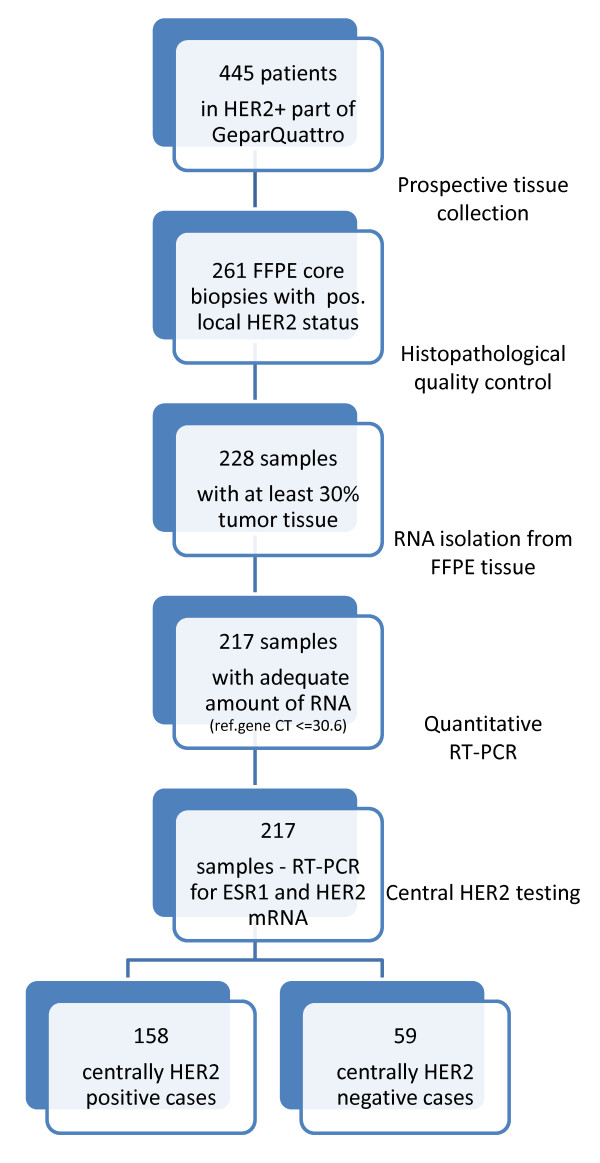
**Consort statement indicating the flow of samples through the study**. HER2, human epidermal growth factor receptor 2; FFPE, formalin-fixed paraffin-embedded; Ct, cycle threshold; ESR1, estrogen receptor 1; RT-PCR, real-time polymerase chain reaction.

The consort statement and clinical characteristics are shown in Figure 1 and Table 1. A total of 261 samples were prospectively collected, of which 33 contained less than 30% tumor tissue. RNA isolation was possible in 217 cases (95.1% of 228). For 158 tumors (72.8% of 217) the local HER2-positive status was confirmed. The remaining 59 tumors (27.2%) were centrally HER2-negative (cHER2-negative status) despite a locally positive HER2 status. Interestingly, the discordance rate was significantly higher in ESR1-positive tumors (34.5%) than in ESR1-negative tumors (18.4%, *P *= 0.009, two-sided Fisher´s test).

**Table 1:** Clinico-pathological data of the study cohort

Characteristic	All cases, number (%)	Centrally evaluated HER2- positive cases, number (%)	Centrally evaluated HER2- negative cases, number (%)
**Samples, number**	217	158	59

**Local HER2 status **(IH/ISH)			
positive	217 (100)	158 (100)	59 (100)
negative	0 (0)	0 (0)	0 (0)
**Central HER2 status **(IH/SISH)			
Positive	158 (72.8)	158 (100)	0 (0)
Negative	59 (27.2)	0 (0)	59 (100)
**HER2 protein expression **(central IH)			
3+	144 (66.4)	144 (91.1)	0 (0)
2+	20 (9.2)	8 (5.1)	12 (20.3)
0/1+	48 (22.1)	2 (1.3)	46 (78.0)
missing	5 (2.3)	4 (2.5)	1 (1.7)
**HER2 gene amplification **(central SISH)			
SISH positive (>2.2)	109 (50.2)	109 (69.0)	0 (0)
SISH negative (<1.8)	49 (22.6)	10 (6.3)	39 (66.1)
SISH equivocal (1.8-2.2)	8 (3.7)	6 (3.8)	2 (3.4)
missing	51 (23.5)	33 (20.9)	18 (30.5)
**HER2 mRNA expression **(central qPCR)			
Positive (>19.5)	141 (65.0)	137 (86.7)	4 (6.8)
Negative (≤19.5)	76 (35.0)	21 (13.3)	55 (93.2)
**ER/PR Status **(local IH)			
ER+ and/or PR+	125 (57.6)	84 (53.2)	41 (69.5)
ER-/PR-	92 (42.4)	74 (46.8)	18 (30.5)
**ESR1 mRNA expression **(central qPCR)			
Positive (>13.8)	109 (50.2)	70 (44.3)	39 (66.1)
Negative (≤13.8)	108 (49.8)	88 (55.7)	20 (33.9)
**Tumor grade**			
G1 to G2	114 (55.1)	78 (49.4)	36 (61.0)
G3	93 (44.9)	72 (45.6)	21 (35.6)
Missing	10 (4.6)	8 (5.1)	2 (3.4)
**Clinical tumor stage**			
cT1 to cT2	155 (71.4)	110 (69.6)	45 (76.3)
cT3 to cT4	62 (28.6)	48 (30.4)	14 (23.7)
**Clinical nodal status**			
cN0	83 (38.2)	61 (38.6)	22 (37.3)
cN+	134 (61.8)	97 (61.4)	37 (62.7)
**Pathological response**			
No pCR	131 (60.4)	84 (53.2)	47 (79.7)
pCR (ypT0/is ypN0)	86 (39.6)	74 (46.8)	12 (20.3)
**Age group**			
≤ 50 years	109 (50.2)	84 (53.2)	25 (42.4)
> 50 years	108 (49.8)	74 (46.8)	34 (57.6)
**Tumor type**			
Ductal/other	207 (95.4)	156 (98.7)	51 (86.4)
Lobular	10 (4.6)	2 (1.3)	8 (13.6)

HER2, human epidermal growth factor receptor 2; SISH, silver *in situ *hybridization; IH: immunohistochemistry; ISH: *in situ *hybridization; qPCR, quantitative polymerase chain reaction; ypT: tumor stage after neoadjuvant therapy; ypN: nodal status after neoadjuvant therapy.

### Concordance of different methods for determination of HER2 to predict response to neoadjuvant trastuzumab/chemotherapy

Additional analyses were performed to validate cHER2 assessment. In addition to the current definition of a positive HER2 status (IHC3+ or IHC2+/SISH >2.0), we used HER2 IHC alone, HER2 SISH ratio, and HER2 SISH copy number, as well as HER2 qRT-PCR. For HER2 mRNA the positive concordance with the current diagnostic standard was 86.7%, the negative concordance was 93.2% and the total concordance was 88.5% (192 of 217 cases, Table 1).

**Figure 2:**
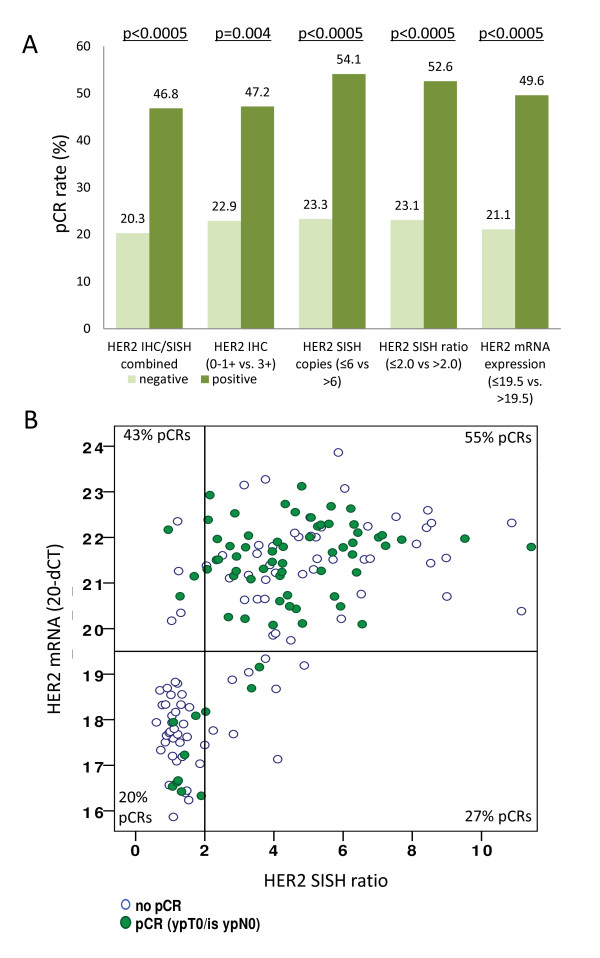
**Comparison of different methods for central assessment of human epidermal growth factor receptor 2 (HER2) overexpression**. (**A**) Five different approaches were used: IHC 3+ or IHC2+/SISH >2.0 (gold standard), HER2 IHC, HER2 SISH ratio, HER2 SISH copy numbers as well as quantitative RT-PCR for evaluation of HER2 mRNA levels. In all approaches the pCR rate was significantly higher in the centrally HER2-positive cases. (*P *= 0.004, for IHC alone, *P *<0.0005 for all other comparisons, two-sided Fisher's exact test). (**B**) Comparison of quantitative HER2 mRNA levels and SISH ratio; pCR cases are marked as dark spots. A total of 11 cases were negative for HER2 mRNA despite an amplification of HER2 by SISH (lower right corner). These cases showed a pCR rate of only 27.3% (three pCRs out of eleven cases). IHC, immunohistochemistry; SISH, silver *in situ *hybridization; RT-PCR, real-time polymerase chain reaction; pCR, pathological complete response, ypT: tumor stage after neoadjuvant therapy.

As shown in Figure 2A, the pCR rate was significantly higher in the cHER2-positive cases. cHER2-positive cases, defined by either IHC/SISH combined, IHC alone, SISH copy number or SISH ratio, had pCR rates of 47%, 47%, 54% and 53%, while the cHER2-negative cases had pCR rates of only 20 to 23% (*P *= 0.004, for IHC alone, *P *<0.0005 for all other comparisons, two-sided Fisher's test) (Figure 2). For HER2 mRNA the pCR rate was 49.6% for the mRNA-positive group and 21.1% for the mRNA-negative group (*P *<0.0005, two-sided Fisher´s test) (Figure 2).

High concordance of 89.2% between HER2 mRNA levels and the SISH ratio was observed (Figure 2B, positive concordance: 90.4%, negative concordance: 86.5%). Those patients who were HER2-negative by both methods had a pCR rate of only 20%. A total of 11 cases were negative for HER2 mRNA despite positive SISH (lower right corner in Figure 2B). Five of those eleven tumors were HER2 IHC 2+ and six were HER2 IHC 3+. These cases showed a comparably low pCR rate of only 27.3% (three pCRs in eleven cases). Interestingly, those seven tumors that were mRNA-positive but SISH-negative had a pCR rate of 43%. Four of those seven tumors were HER2 3+ by IHC, while two were HER2 2+ and one was HER2 0 to 1+.

For comparison of HER2 mRNA levels and SISH copy number, concordance of 91.1% was achieved. For hormone receptor (HR) status, 185 of the 217 cases were concordant regarding the comparison of ER IHC and ESR1 mRNAs, resulting in a concordance of 85.2%.

### Quantitative assessment of ESR1 and HER2 mRNA levels

**Figure 3:**
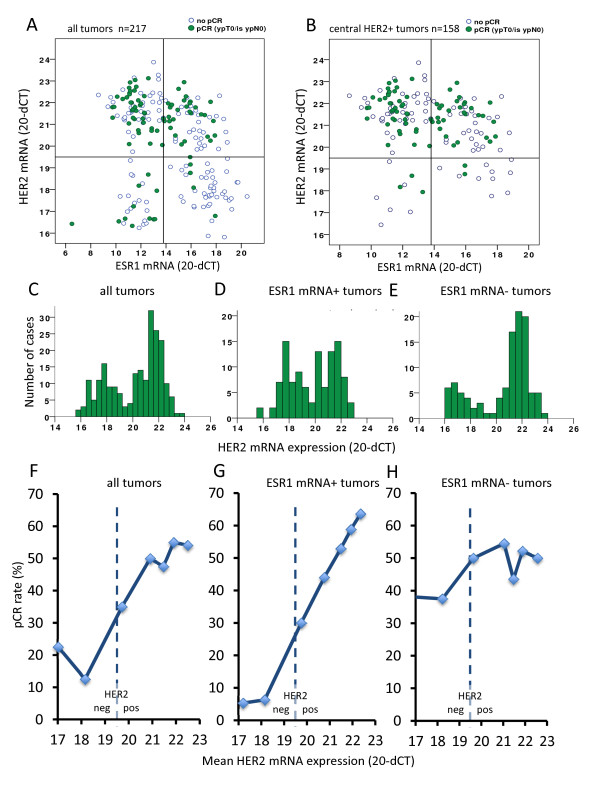
**mRNA-based combined quantitative determination of human epidermal growth factor receptor 2 (HER2) and estrogen receptor 1 (ESR1) mRNA**. Cases with a pCR are marked as dark spots. (**A**) Total of 217 cases. (**B**) Centrally HER2-positive cases (*n *= 158). In this group a total of 21 cases (13.3%) were HER2-negative by mRNA analysis; the pCR rate of those 21 cases was only 23.8%. The pCR rate in those 137 cases that were HER2-positive by mRNA analysis was 50.4%, (*P *= 0.03, two-sided Fisher's exact test). (**C, D, E**) All 217 cases: different distribution patterns of HER2 mRNA with a continuous distribution in ESR1-positive tumors (**D**) and a more dichotomous distribution in ESR1-negative tumors (**E**). STEPP analysis for all 217 tumors (**F**), ESR1-positive tumors (**G**) and ESR1-negative tumors (**H)**. HER2 mRNA levels were linked to pCR rate in the group of ESR1-positive tumors with a continuously increased pCR rate. pCR, pathological complete response; STEPP, subpopulation treatment effect pattern plot.

The combined quantitative determination of HER2 and ESR1 mRNA with predefined cutoffs resulted in four separate groups (Figure 3). If the analysis was restricted to the 158 cHER2-positive cases (Figure 3B), a total of 21 cases (13.3%) were HER2-negative by mRNA analysis. The pCR rate of these 21 cases was 23.8% (Figure 3B). In contrast, the pCR rate was significantly higher in those 137 patients who were cHER2-positive and HER2 mRNA-positive (pCR rate 50.4%, *P *= 0.03, two-sided Fisher's test).

### Separate evaluation of ESR1-positive and -negative tumors by STEPP analysis and logistic regression

The distribution of HER2 mRNA expression is different in ESR1-positive and ESR1-negative cases. For ESR1-negative cases HER2 mRNA has a typical bimodal distribution (Figure 3E). In contrast, for ESR1-positive tumors HER2 mRNA levels are more continuously distributed indicating a continuum of HER2 expression in this group (Figure 3D). This type of different distribution was not observed for the HER2 SISH ratio.

**Figure 4:**
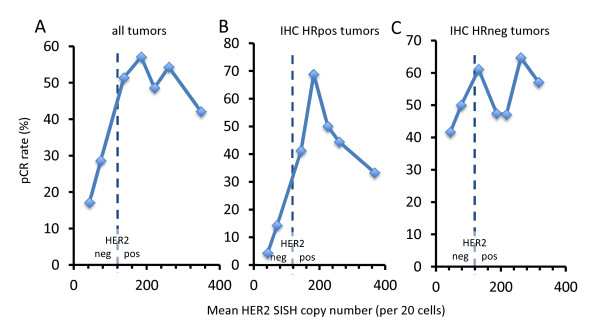
**Subpopulation treatment effect pattern plot (STEPP) analysis using the classical parameters human epidermal growth factor receptor 2 (HER2) silver *in situ *hybridization (SISH) copy number and hormone receptor immunohistochemistry**. Shown are analysis for all tumors (**A**), hormone receptor-positive tumors (**B**) and hormone receptor negative tumors (**C**). In contrast to the data shown ins the classical parameters were not linked to pCR rate. pCR, pathological complete response; IHC, immunohistochemistry; HR, hormone receptor.

To evaluate the impact of different levels of HER2 mRNA on pCR in the groups of ESR1-positive and -negative tumors, we performed STEPP analysis. As shown in Figure 3G, HER2 mRNA levels were significantly linked to pCR rate in the group of ESR1 mRNA-positive tumors: in this group, the pCR rate continuously rose with increased HER2 mRNA levels. In contrast, for ESR1 mRNA-negative tumors an abrupt increase of the pCR rate occurred once the cutoff between HER2-positive and -negative tumors was crossed, while HER2 mRNA levels did not further influence the pCR rate once a tumor was in the HER2-positive group (Figure 3H). A similar assessment using the traditional factors, HR status by IHC and SISH copy number, did not show a rise of pCR rates with increased SISH ratios in HR-positive tumors (Figure 4).

**Figure 5:**
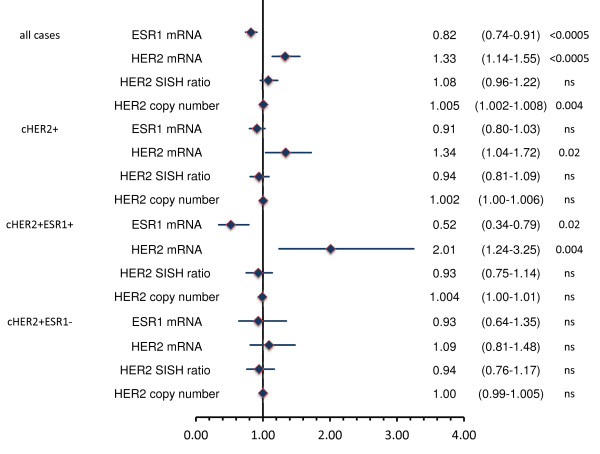
**Odds ratios for pathological complete response (pCR) for the quantitative markers estrogen receptor 1 (ESR1) mRNA (per dCT), human epidermal growth factor receptor 2 (HER2) mRNA (per dCT), silver *in situ *hybridization (SISH) ratio and SISH copy number in different subgroups**. Results in the three columns (right) represent as odds ratios, 95% CI and *P*-values. ESR1 mRNA was significant only in the group of all 217 cases (which also contained the centrally evaluated HER2 (cHER2)-negative cases). If the analysis was restricted to the centrally evaluated HER2-positive cases, ESR1 mRNA was not significant. HER2 mRNA levels were only significant in the group of ESR1-positive tumors, similar to the subpopulation treatment effect pattern plot (STEPP) analysis. In contrast to the mRNA analysis, HER2 SISH ratio and copy number were not significant in centrally evaluated HER2-positive cases. ns, not signficant.

In a parallel approach, odds ratios for pCR for the three quantitative markers, ESR1 mRNA, HER2 mRNA and SISH ratio, were determined in different subgroups. The predictive effect of HER2 mRNA levels was particularly strong in the group of cHER2-positive/ESR1-positive tumors; in this group ESR1 mRNA level was also significant (Figure 5). In contrast, HER2 as well as ESR1 mRNA levels were not significant in cHER2-positive/ESR1-negative tumors, similar to the results of the STEPP analysis. If the analysis was restricted to cHER2-positive cases, ESR1 mRNA was a significant predictive marker only in the groups of ESR1-positive tumors. Neither SISH ratio (Figure 5) nor SISH copy number (Figure 5), was significant in the group of cHER2-positive cases.

## Discussion

Our investigation shows that in HR-positive tumors the response to neoadjuvant treatment with trastuzumab plus anthracyline-taxane chemotherapy is driven by the degree of HER2 mRNA expression. This phenomenon could not be observed in the HR-negative subset. Interestingly, Soon Paik's group has described a similar finding in the adjuvant setting. While their full paper is not published yet, a summary of their results has been included in the recent St Gallen recommendations as follows: 'An interesting STEPP analysis from the adjuvant trastuzumab NSABP B-31 trial examined the degree of HER2 mRNA expression and corresponding trastuzumab benefit separately for patients with estrogen receptor-positive and estrogen receptor-negative disease. The striking finding was that among patients with estrogen receptor-positive disease, trastuzumab benefit in terms of 8-year disease-free survival was entirely confined to those with the higher levels of HER2 mRNA expression' [15].

Similar to these findings in the adjuvant setting, there is a considerable difference in our neoadjuvant study between ESR1-positive/HER2-positive and ESR1-negative/HER2-positive tumors. For ESR1-negative/HER2-positive tumors the amount of HER2 mRNA is not further relevant for response once a tumor is in the HER2-positive group. mRNA levels of HER2 have a dichotomous distribution and HER2 can be used as a categorical parameter in this group.

For ESR1-positive/HER2-positive tumors the situation is different; HER2 mRNA has a more continuous distribution and the response to neoadjuvant trastuzumab/chemotherapy rises continuously with the amount of HER2 mRNA within the HER2-positive tumor group. This suggests that those luminal tumors with higher activity of the HER2 pathway (measured as increased mRNA levels) are more dependent on this pathway and thus more responsive to trastuzumab targeted therapy. This finding is supported by the STEPP analysis and we observed the same effect with the classical approach of logistic regression, which also showed a significant effect of HER2 mRNA levels (measured as a continuous variable) on pCR only in the ESR1-positive/HER2-positive group. The traditional method of HER2 SISH ratio or copy number was not able to provide a similar result by STEPP or logistic regression analysis, similar to the finding in the adjuvant HERA trial [27].

The relevance of crosstalk between the ER pathway and the HER2 pathway has been described in several *in vitro *cell culture and animal models [14,28,29], *Amplified in breast *(*AIB*)-*1 *[30,31] as well as *Paired box gene 2 *(*PAX2*) [32] have been identified as relevant mediators of this crosstalk.

The hypothesis derived from those investigations and our results would be that two important growth factor pathways significantly influence ESR1-positive/HER2-positive tumors and either HER2 or ER may be the driver of cell proliferation and survival. With sustained HER2 inhibition ER could function as a key escape or survival pathway, which may result in resistance to trastuzumab. However when HER2 mRNA expression is very high the primary driver of proliferation may still be the HER2 pathway even in the presence of the activated ER pathway. These findings are consistent with two neoadjuvant trials where significantly lower pCR rates were observed in ER-positive/HER-positive tumors compared to ER-negative/HER2-positive disease [7,8]. However, in a recently reported neoadjuvant trial, response rates to anti-HER2 treatment with lapatinib and trastuzumab (without chemotherapy) were fairly high (pCR 21%) when combined with endocrine treatment if HRs were present [33]. As in the adjuvant setting trastuzumab or lapatinib therapy (in contrast to the neoadjuvant approach) is usually combined with endocrine therapy in the HR-positive group; the combined inhibition of both pathways is already used in clinical practice [34,35]. It would be interesting to further evaluate the contribution of the endocrine therapy to outcome in ESR1-positive/HER2-positive tumors.

Another finding of our study is the rather high rate of discordance of 27% between central and local evaluation of HER2. We have validated this finding by the use of different methods for central pathology. A similar rate of 20% inaccurate HER2 measurements has been reported before [9] based on results of the NSABP [36] and the N9831 study [37,38]. Discordance has also been observed between different reference laboratories, in particular for borderline cases [39]. Interestingly, in our study the discordance was higher in ESR1-positive tumors, which might be partially due to the more continuous distribution of HER2 mRNA in this group. Pinhel *et al*. [40] have also observed this different distribution of HER2 mRNA in ER-positive vs -negative tumors in a recent report.

The high level of false-positive cases gave us the possibility to evaluate the response to trastuzumab in HER2-negative tumors. Centrally HER2-negative tumors had a pCR rate of only 20%, which is in the range of the pCR rate of HER2-negative tumors in the GeparTrio trial (17.6%). This validates previous findings that HER2 is the crucial biomarker for trastuzumab-based therapy. However, it differs from an analysis of the NSABP-B31 suggesting a benefit of adjuvant trastuzumab even in cHER2-negative tumors [41]. There are two main differences between NSABP-B31 and our study: we used the neoadjuvant setting, which allowed us to directly study response in the primary tumor, but we could not evaluate the effects on micro-metastases as well as the contribution of adjuvant endocrine therapy. Furthermore, the rate of cHER2-negative cases in NSABP-B31 was only 9.7%, compared to 27% in our study. It would be very interesting to await the results of the NSABP B47 [42], which is currently evaluating the benefit of trastuzumab in low HER2-expressing tumors.

As an additional method we have evaluated HER2 mRNA expression by qRT-PCR. Recently, Dabbs *et al*. found that HER2 mRNA levels were negative by recurrence score in 10 (42%) of 24 HER2-positive cases [43]. The same finding had already been shown in 638 samples from the NSABP-B31 with an overlap of HER2 mRNA expression levels between HER2-positive and -negative tumors [40]. In our study 11 cases were negative for HER2 mRNA despite positivity by SISH, and those cases had a low pCR rate of only 27%. Furthermore seven tumors were mRNA-positive but SISH-negative with a pCR rate of 43%. Therefore, in our small cohort there is no evidence that patients with central IHC-positive results but negative HER2 mRNA have relevant benefit from trastuzumab with regard to pCR, which raises the hypothesis that HER2 mRNA might be more suitable for response prediction than SISH. We would like to emphasize that the low number of cases makes it impossible to fully evaluate this hypothesis in the context of our study, and that currently all indications for HER2-targeted therapies should be based on the established Food and Drug Administration (FDA) criteria.

The differences between HER2 mRNA and the classical methods of IHC and SISH might explain the finding that a different magnitude of benefit according to ER status has not been seen for trastuzumab in any of the adjuvant trastuzumab trials.

There are several limitations of our study; it was retrospective, the analysis could only be performed in a subpopulation, and we used only one pCR definition without separating the group of residual ductal carcinoma in-situ (DCIS). This separation was not possible in our cohort due to the smaller sample size. It should also be noted that some studies suggest that pCR might not be a reliable surrogate for long-term disease outcome in ER+/HER2+ disease [44]. The advantages of the study are that we used a population from a prospective clinical trial with a standardized assay system, as well as a predefined hypothesis and analysis plan.

## Conclusions

In summary, our results provide further evidence for the concept that HER2-positive/non-luminal and ESR1-positive/HERpositive tumors are different biological entities. Several randomized trials have shown that the benefit of adjuvant trastuzumab is significant in the cohort of HER2-positive tumors as well as in subgroups based on HR expression. It would be very interesting to evaluate HER2 mRNA levels in this context, since HER2 mRNA expression may select those ER-positive/HER2-positive tumors with an optimal benefit from trastuzumab. Another important issue would be to evaluate HER2 mRNA levels for response to different types of HER2 targeted agents, for example, lapatinib [45,46] or pertuzumab [47]. Interestingly, a recent analysis in the NSABP B-41 trials has suggested differences between lapatinib, trastuzumab and their combination depending on the protein expression level of HER2 [48]. Additional evaluations are planned in the GeparQuinto and the GeparSixto trials of the AGO B and the German Breast Group within the European FP7 project, RESPONSIFY.

## Abbreviations

AIB: amplified in breast; cHER2: centrally evaluated HER2 status; Ct: cycle threshold; ER: estrogen receptor; ESR1: estrogen receptor 1; FDA: Food and Drug Administration; FFPE: formalin-fixed paraffin-embedded; GOI: genes of interest; HER2: human epidermal growth factor receptor 2; HR: hormone receptor; IHC: immunohistochemistry; NSABP: National Surgical Adjuvant Breast and Bowel Project; PAX2: Paired box gene 2; pCR: pathological complete response; qRT-PCR: quantitative real-time polymerase chain reaction; REMARK: reporting recommendations for tumor marker prognostic studies; SISH: silver *in situ *hybridization; STEPP: subpopulation treatment effect pattern plot; TMA: tissue-microarray.

## Competing interests

CD and RK are shareholders of Sividon Diagnostics. CD has received research funding from Siemens Healthcare. CD and JH have received research funding from GSK. All other authors declare no competing interests.

## Authors' contributions

CD, JH, SL, RK, GvM and MU have designed the study and participated in data acquisition, analysis and interpretation as well as manuscript writing. KM, SD-E, JCB, BVS and JP participated in the statistical analysis and data acquisition as well as manuscript writing. CS, PAF, BVS, KE, MR, M-LH and HT participated in the acquisition of data as well as writing and revision of the manuscript. All authors have read and approved the manuscript for publication.
